# Assessment of the Nutraceutical Properties of Wild Strawberry (*Fragaria vesca* L.) Extracts on Human Colorectal Cell Lines

**DOI:** 10.1002/mnfr.70018

**Published:** 2025-04-10

**Authors:** Monica Oldani, Lorenzo Guzzetti, Emiliano Pioltelli, Davide Sala, Davide Panzeri, Maura Brioschi, Matilde Forcella, Paola Fusi

**Affiliations:** ^1^ Department of Biotechnology and Biosciences University of Milano‐Bicocca Milan Italy; ^2^ Integrated Models for Prevention and Protection in Environmental and Occupational Health, (MISTRAL) Interuniversity Research Center Italy

**Keywords:** colorectal cancer, functional food, nutraceuticals, prevention, strawberry

## Abstract

Colorectal cancer is the third most common cancer worldwide; its higher incidence in the Western world, compared to rural areas of Africa and Asia, led to its classification among the so‐called Westernized diseases. The wild strawberry *Fragaria vesca* L. is endowed with several bioactive components, such as polyphenols, vitamins, terpenes, and organic acids that can contribute to cancer prevention. In this study, we chemically characterized a wild strawberry extract through high‐resolution mass spectrometry and evaluated its antioxidant properties on two human colorectal cancer cell lines: KRAS mutated SW480 cells and E705 cells. We found that treatment with the extract induced cell cycle arrest in the G2 phase in SW480 cells, while it led E705 cells to apoptosis through a significant increase in the reactive oxygen species level. Wild strawberry extract is a promising dietary supplement for both wild‐type and KRAS‐mutated patients who exhibit a more aggressive cancer phenotype. In addition, the lack of toxicity of wild strawberry extract toward healthy colorectal cells makes this food a promising chemopreventive nutritional supplement.

## Graphical abstract

The study explores the nutraceutical potential of wild strawberry (*Fragaria vesca L*.) by assessing its effects on multiple colorectal carcinoma cell lines. Wild strawberry phytochemicals were able to reduce SW480 cell viability by inducing cell cycle arrest and by promoting programmed cell death in E705 cells, with no toxicity on healthy colorectal cells. These findings support *F. vesca* as a potential functional food and promising chemopreventive supplement.

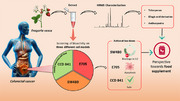

## 1 Introduction

Globally, colorectal cancer (CRC) is the third most common cancer with about one million new cases yearly, and is the second deadliest after lung cancer [1]. The occurrence of this cancer does not seem to have any relationship with the age, sex, or ethnicity of patients. However, epidemiological studies have found a possible correlation between specific geographical areas and a higher risk of developing CRC, leading this cancer to be added to the list of the so‐called “Westernized” diseases [2]. The incidence of CRC is rooted in North America, Australia, New Zealand, and Europe, while it occurs at a much lower rate in rural areas of Africa and Asia [2]. With these premises, it has become increasingly apparent that not only genetic but also environmental factors may be responsible for its occurrence. Some dietary factors, such as alcohol and excessive consumption of red/processed meats, appear to be positively correlated with the development of CRC; conversely, a healthy diet could help prevent it [3]. In addition, diet is estimated to contribute to 20%–42% of all human cancers and 50%–80% of colon cancers [4]. Therefore, the adoption of dietary habits promoting the assumption of foods endowed with a chemopreventive role seems to have many benefits in reducing the risk of developing cancer and other types of noncommunicable diseases [5, 6, 7].

Consumption of vegetables, fruits, and herbs rich in bioactive components (BACs), such as polyphenols, carotenoids, and vitamins, has been indicated as a potential cancer‐preventive factor [8, 9, 10, 11]. Indeed, it has been proven that some BACs can inhibit colon cancer cell proliferation by acting on multiple regulatory pathways, such as IGF, NF‐κB, PI3K/Akt/MAPK, and the Wnt/β‐catenin pathways [10, 12, 13]. Moreover, BACs have also been reported to trigger reactive oxygen species (ROS) overproduction, leading cancer cells to apoptosis [14]. Cancer is one of many diseases associated with mitochondrial impairment and ROS overproduction [15]; however, ROS overproduction in cancer cells must be kept within a beneficial range for cell proliferation, without exceeding the threshold leading to senescence and cell death [16]. Two strategies are therefore possible to counteract cancer cell proliferation: either a further increase in ROS level inducing cell death or its reduction, improving mitochondrial functionality, and inhibiting cell proliferation.

Strawberries are known to be endowed with a wide array of antioxidant and antiinflammatory properties [17]. Specifically, *Fragaria vesca* L., also known as wild strawberry and belonging to the Rosaceae family, contains several BACs [18, 19]. Wild strawberries are not only tasty and aromatic fruits but they are also rich in easily absorbable monosaccharides, vitamins (C, B1, B2, K), and organic acids (malic, citric, and salicylic) [20, 21]. The average concentration of total polyphenols in wild strawberry is even higher than that found in the common strawberry, known as *Fragaria* x *ananassa* L. [21, 22], and a similar pattern has also been found in their antioxidant activity [20, 23]. An anticancer effect of *Fragaria* x *ananassa*, the most common strawberry species available on the market, has already been suggested [24, 25]. In this study, we aim at evaluating the nutraceutical potential of the BACs extracted from wild strawberries in human colorectal carcinoma cell lines. The extracts are also characterized through mass spectrometry‐based approaches to determine the identity of the main BACs occurring in them, in addition to the content of free sugars and amino acids. The final goal of the study is to evaluate the potential anticancer activity of wild strawberry BACs to design novel functional foods that may be adopted in strategies of nutritional prevention.

## 2 Experimental Section

### 2.1 Chemical Characterization of Wild Strawberry Extracts

#### 2.1.1 Sample Collection

Wild strawberry plants were cultivated at the Council for Agricultural Research and Economics (CREA) in Sanremo, Italy. Before the extraction, the harvested strawberries were freeze‐dried for 48 h (Sp Scientific, BenchTopPro, USA) to completely remove their water content. After the freeze‐drying process, strawberries were ground using a laboratory grinder (IKA, Germany) to obtain a fine powder and stored at −20°C before being analyzed.

#### 2.1.2 Sample Extraction and SPE‐Mediated Fractionation

The extraction process was carried out by a hydro‐alcoholic solvent (70% MeOH v/v, pH 3.5) with a drug‐to‐solvent ratio of 1:20 w/v. After the addition of the extraction solvent, the samples were agitated on an orbital shaker (Asal 711, Italy) at 30 rpm for 15 min and then centrifuged at 5000 *g* for 5 min. The supernatant was collected, while the pellet underwent a second extraction cycle to completely deplete the matrix. The extracts obtained were dried using a rotary evaporator (Steroglass, Italy). All the extracts were stored at −20°C for subsequent analysis. The extracts were purified by solid phase extraction (SPE) using Strata X 33 µm polymeric reversed phase 30 mg/3 mL cartridges (Phenomenex, USA), which were loaded with the extracts (at a concentration of 20 mg/mL) and then eluted with a gradient spanning from 10% to 100% MeOH + 0.1% HCOOH v/v. The eluted fractions were kept at −80°C for the subsequent chemical analyses. Both the total extract, the flowthrough, and the eluted fraction were characterized for the content of secondary compounds, free sugars, and free amino acid composition. Furthermore, each sample was characterized by high‐resolution mass spectrometry (HRMS).

#### 2.1.3 Quantitative Analysis of the Sugars Content

The total free sugar content (TFSC) in the extracts was assessed using the enzymatic kit provided by Megazyme, Ireland as described in Pioltelli et al. [26].

#### 2.1.4 Determination of Total Phenolic and Flavonoids Content and Antioxidant Activity

Three different colorimetric assays accessed the analysis of the phytochemical content of the different strawberry fractions: Folin–Ciocalteau assay to evaluate the total phenol content (TPC), the diphenyl‐picrylazil (DPPH) assay to measure the Trolox equivalent antioxidant capacity (TEAC), and the aluminum chloride (AlCl_3_) assay to evaluate the total flavonoid content (TFC), as described in Pioltelli et al. [26].

#### 2.1.5 Quantitative Analysis of Aminoacid Content

The content of free amino acids occurring in the samples was analyzed by HPLC‐UV‐FLD (Infinity 1260, Agilent, USA), using o‐phtalaldehyde (OPA) and fluorenylmethoxycarbonyl (FMOC protecting group) as precolumn derivatizing agents. The chromatographic column was a Kinetex C‐18 (2.1 × 100 mm, 2.6 µm; Phenomenex, USA), and the mobile phases were the following: (A) NH_4_COOH 5 mM pH 7.9; (B) MeCN/MeOH/H_2_O 45:45:10 v/v/v. The flow rate was 0.4 mL/min. The chromatographic conditions were set as follows: 0–13.4 min from 2% B to 57% B, 13.4–13.5 min from 57% B to 100% B, and held at 100% B until 18 min. Only for the analysis of tryptophan (which did not require any derivatization), the chromatographic gradient was slowed down and set as follows: 0–10 min from 2% B to 10% B, then the column was washed at 100% B for 5 min and reconditioned at 2% B for 5 min. Column temperature was set at 40°C. The precolumn derivatization was performed as follows: 5 µL borate buffer pH 10.1 (Agilent, USA); 1 µL sample/calibration point/blank; 1 µL OPA mixture (1 mg/mL in 2% v/v 2‐mercaptoethanol) or Fmoc (2.5 mg/mL in MeCN); and 3 µL of injection diluent (CH_3_COOH 1 M). The injection volume was 10 µL. The detection through the FLD was made as follows: for FMOC derivatized amino acids (proline, valine, methionine, and arginine): excitation at 262 nm and emission at 325 nm and for OPA derivatization of amino acids (all but tryptophan): excitation at 338 nm and emission at 450 nm. Tryptophan was detected without derivatization (excitation at 280 nm and emission at 350 nm). For each amino acid, a calibration curve was produced ranging from 80 ng/mL to 2 µg/mL.

#### 2.1.6 Characterization of the Phytochemical Content by LC‐UV/PDA‐HRMS Analysis

The phytochemical composition of each fraction was analyzed by HRMS through an Acquity UPLC HClass system (Waters, USA), equipped with a PDA detector and coupled to a G2‐Xevo‐qToF mass spectrometer (Waters Corporation, Milford, USA). The analytes were separated by a reverse phase column (Zorbax SB‐C_18_, 2.1 × 100 mm; 3.5 µm, Agilent, USA) using H_2_O + 0.1% HCOOH and MeCN + 0.1% HCOOH as mobile phases, and the column temperature was set at 30°C. Samples were analyzed at a concentration of 200 µg/mL injecting 10 µL each. The mass spectrometer was set to acquire mass spectra in MS^e^ mode, to account both for the full scan traces at 6 eV collision energy and for the main fragments, with a ramp of collision energy from 15 to 40 eV. Both the negative and positive ion currents were detected. The lockmass (LeuEnk 200 pg/µL) was infused at 10 µL/min and acquired every 2 s. The capillary voltage was set at 1.0 kV in both ion modes. The source temperature was set at 140°C, and the desolvation temperature was 600°C. The analytes were characterized by both literature research and natural products libraries. In particular, UNIFI (ver. 1.9.4.053), MS‐DIAL (ver. 5.1.230912) [27], and MS‐FINDER (ver. 3.60) software were used to characterize the occurring metabolites.

To obtain an estimate of the amounts of the identified components, the PDA detector wavelength (*λ*) was set to the range 190–500 nm, and three channels were set to 255, 365, and 475 nm to monitor the UV/visible absorbing constituents of the extract. In particular, the following standards purchased from Merck (Germany) were used: pinocembrin (optimal *λ* 290 nm), gentiopicroside (optimal *λ* 274 nm), taxifolin (optimal *λ* 288 nm), liquiritin (optimal *λ* 288 nm), ellagic acid (optimal *λ* 255 nm), cyanidin‐3‐*O*‐glucoside (optimal *λ* 500 nm), kaempferol (optimal *λ* 365 nm), catechin (optimal *λ* 280 nm), peonidin‐3‐*O*‐glucoside (optimal *λ* 500 nm), coumaric acid (optimal *λ* 309 nm), gallic acid (optimal *λ* 270 nm), and pelargonidin (optimal *λ* 280 nm). A calibration curve ranging from 100 ng/mL to 20 µg/mL was set up for each standard reference. Concentrations are expressed as milligram equivalent/gram dry extract.

### 2.2 Cell Cultures

CCD 841 CoN (ATCC CRL‐1790) human healthy mucosa cell line was grown in EMEM medium supplemented with heat‐inactivated 10% fetal bovine serum (FBS), 2 mM l‐glutamine, 0.1 mM nonessential amino acids, 100 U/mL penicillin, and 100 µg/mL streptomycin. SW480 (ATCC CCL‐228) and E705 (kindly provided by Fondazione IRCCS Istituto Nazionale dei Tumori, Milan, Italy) human colorectal cancer cell lines were grown in RPMI 1640 medium supplemented with heat‐inactivated 10% FBS, 2 mM l‐glutamine, 100 U/mL penicillin, and 100 µg/mL streptomycin. All cell lines were maintained at 37°C in a humidified 5% CO_2_ incubator. ATCC cell lines were validated by short tandem repeat profiles that are generated by simultaneous amplification of multiple short tandem repeat loci and amelogenin (for gender identification). All the reagents for cell cultures were supplied by EuroClone (EuroClone S.p.A, Milan, Italy).

### 2.3 Viability Assay

CCD 841, SW480, and E705 cell lines were seeded in 96‐well microtiter plates at a density of 1 × 10^4^ cells/well, cultured in a complete medium for 24 h and then exposed to different concentrations of three wild strawberry extracts. The total extract was tested at concentrations ranging from 0.01 up to 2 mg/mL, the flowthrough from 0.1 to 1 mg/mL, and at last, the eluate was assayed between 0.05 and 0.4 mg/mL. After 48 h at 37°C, the medium was replaced with a complete medium without phenol red containing 10 µL of 5 mg/mL MTT (3‐(4,5‐dimethylthiazol‐2‐yl)‐2,5‐diphenyltetrazolium bromide) and, based on the metabolic differences between healthy and tumor cells, CCD 841 cell line was incubated for 4 h and the two tumor cell lines for 2 h at 37°C. At the end of the scheduled time, the formazan crystals produced by the cells were dissolved in the acid solubilization solution. The MTT solution and the solubilization solution were prepared according to Riss et al. [28]. The absorbance was measured at 570 nm using VICTOR Multilabel Plate Reader (PerkinElmer, Waltham, MA, USA), and the cell viability was expressed as a percentage against untreated cell lines used as controls.

### 2.4 Oxidative Stress Assays

The oxidation of 2′,7′‐dichlorofluorescin diacetate (H_2_DCF‐DA) (Merck KGaA, Darmstadt, Germany) was employed to estimate general ROS production [29]. 1 × 10^5^ cells/well were seeded into a 6‐well culture plate, and 24 h after seeding cells were exposed to the wild strawberry eluate fraction for 48 h. All samples were incubated with 5 μΜ H_2_DCF‐DA dissolved in PBS for 20 min at 37°C. The fluorescence intensity of DCF was detected with the FL‐1 channel and analyzed using CytExpert 2.3 Software (Beckman Coulter, USA).

To assay catalase (CAT), glutathione S‐transferase (GST), glutathione peroxidase (Gpox), glutathione reductase (GR), superoxide dismutase (SOD), and γ‐glutamylcysteine synthetase (GCS) activities, 1 × 10^6^ cells of both tumor cell lines were seeded in 100 mm Ø Petri dishes and 24 h after seeding were exposed to the wild strawberry eluate fraction for 48 h. All homogenates were prepared according to Oldani et al. [30], as well as the protocols for testing the activity of the above‐mentioned enzymes. The GCS activity was assayed according to Huang et al. [31]. The Bradford method [32] was used to calculate the specific activity of each enzyme related to total protein concentration in all samples.

To assay glutathione levels cells were seeded at the same density used for enzyme activity sample preparation; after 48 h treatment, cells were prepared as described in Oldani et al. [33]. The results were expressed as nmol/mg cells.

### 2.5 Apoptosis and Cell Cycle Analysis

E705 and SW480 cells were seeded on a 24‐well plate at a density of 1 × 10^5^ cells/well. Cells, 24 h after seeding, were exposed to the wild strawberry eluate fraction for 48 h and then harvested by trypsinization and centrifugation. Apoptosis was determined using the FITC Annexin V/Dead Cell Apoptosis Kit with FITC annexin V and PI for Flow Cytometry (catalog number V13242, Thermo Fisher Scientific, Waltham, MA, USA) according to the manufacturer's protocol. Cell cycle analysis was performed according to Kim and Sederstrom [34]. Cells were analyzed by flow cytometry (CytoFLEX; Beckman, USA) and using CytExpert 2.3 Software (Beckman Coulter, Inc., Brea, California, USA). The kinetic cell proliferation assay allowed the assessment of cell growth rate over time by microscopic cell counting through the Burker chamber. The assay was performed exclusively on the SW480 tumor cell line treated with the wild strawberry eluate fraction. On the first day, cells were seeded on a 12‐well plate at a density of 2 × 10^5^ cells/well and were counted at three different treatment times (24, 48, and 72 h).

### 2.6 Western Blotting

For Western‐blot analysis, the cells were seeded at a density of 1 × 10^6^ cells/100 mm Petri cell culture dish, 24 h after seeding were exposed to the wild strawberry eluate fraction for 48 h, and then samples were prepared according to Oldani et al. [33]. Homogenates were analyzed for protein content by the BCA protein assay [35]. Standard procedures and materials to carry out SDS‐PAGE and Western blot are described in Oldani et al. [33]. Protein levels were quantified by measuring the bioluminescence obtained after incubation with the ECL detection system (Millipore) and using Scion Image software v. 4.0 (Scion Corp., Frederick, MD, USA). The primary antibodies used were anti‐P‐Cdc2 (catalog number #2543, dilution 1:1000, Cell Signaling Technology, Danvers, MA, USA), and anti‐Vinculin (catalog number # V9131, dilution 1:10 000, Merck KGaA, Darmstadt, Germany). A 1:8000 dilution of IgG‐HRP anti‐rabbit (catalog number #7074) or anti‐mouse (catalog number #7076) conjugated secondary antibodies (Cell Signaling Technology, Danvers, MA, USA) were utilized.

### 2.7 Statistical Analysis

Data obtained from the colorimetric and enzymatic assays were analyzed using ANOVA coupled with the Tukey HSD post hoc test for multiple comparisons to investigate potential variations in the chemical composition of the different fractions of the extracts. Concerning cell analysis, experiments for each concentration were carried out in triplicate. Statistical significance for each treatment was obtained by applying ANOVA, followed by Dunnett's post hoc test to compare the difference existing between the conditions analyzed with respect to the control. All analyses were conducted using the R statistics programming environment (version 4.3.1). Graphs were obtained by using GraphPad Prism Software v. 6.01. The densitometric analysis was performed by using Scion Image software v. 4.0 (Scion Corp., Frederick, MD, USA).

## 3 Results

### 3.1 Phytochemical Analysis of Wild Strawberry Extracts and Subfractions

Table 1 shows the results of the colorimetric and enzymatic assays performed on the different fractions of the extract. As shown, wild strawberry total extract was characterized by a high TSFC, which was not retained by the SPE cartridge and was therefore collected mainly in the flow‐through fraction. Conversely, the TPC, TEAC, and TFC sample values were significantly higher in the eluted fraction, indicating that the cartridge retained most of the BACs. In Table S1, the quantification of the free amino acids occurring within the different fractions is reported. Results showed that most of the amino acids, particularly asparagine and glutamine, were accumulated within the eluted fraction. In the eluted fraction, a total amino acid content of about 20 µg/mg was detected, while in the flowthrough and in the total extract these compounds were significantly lower, with concentrations of 4.2 and 1.4 µg/mg, respectively.

**TABLE 1:** Phytochemicals and sugar content of wild strawberry total extracts, flowthrough, and eluate fractions.

	TPC (µg GAE/mg)	TEAC (µmol TE/g)	TFC (µg QE/mg)	TFSC (µg free sugars/mg)
Total extract	34.56 ± 3.96^a^	339.55 ± 27.89^d^	23.72 ± 2.58^g^	537.21 ± 61.77^l^
Flowthrough	9.23 ± 1.02^b^	82.29 ± 9.53^e^	3.12 ± 0.53^h^	625.73 ± 78.09^l^
Eluate	445.39 ± 39.22^c^	3997.78 ± 337.93^f^	327.82 ± 29.23^i^	193.5 ± 11.45^2^

*Note*: Values are reported as mean ± SEM. Means identified by a different letter are significantly different among each other (*p* < 0.05 from ANOVA followed by Tukey post hoc test).

Abbreviations: TEAC = trolox equivalent antioxidant capacity, TFC = total flavonoid content, TFSC = total free sugar content, TPC = total phenol content.

**FIGURE 1:**
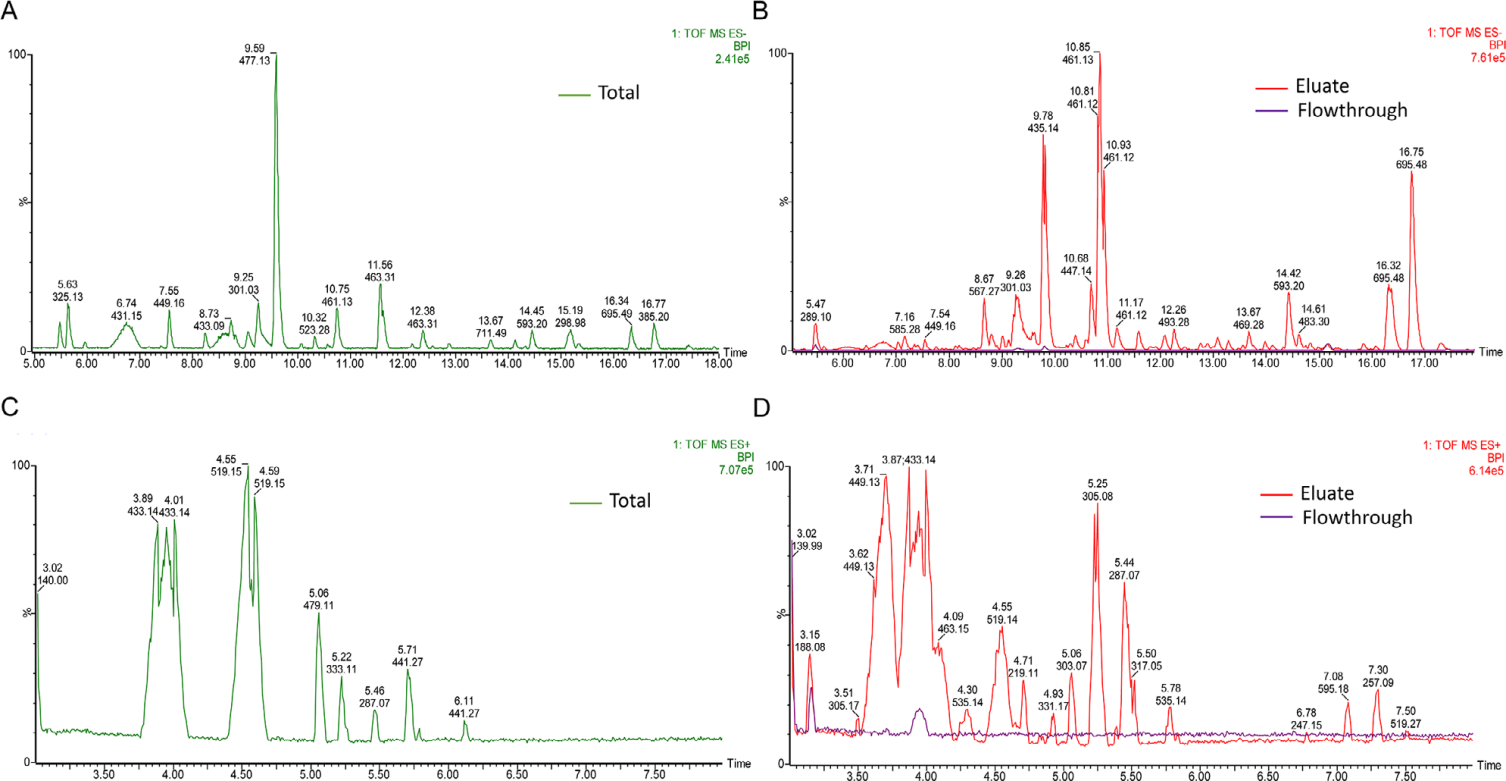
BPI chromatograms of the full scan traces of wild strawberry total extract (A, C), eluate and flowthrough (B, D) in negative and positive ion mode, respectively.

The chemical identity of the BACs occurring in the sample is disclosed in Table S2, together with their estimated abundances, and the chromatograms of the three analyzed fractions in both positive and negative currents are reported in Figure 1. Most BACs identified are ellagic acid derivatives, such as ellagitannins, anthocyanins, flavonoids, procyanidins, phenolic acid derivatives, and terpenes. As reported in Figure 1, these compounds, occurring in the total extract, were recovered mainly in the eluted fraction as confirmed by the extracted ion chromatogram (EIC) intensities of the components reported in Table S2.

### 3.2 Wild Strawberry Eluted Fraction Shows Anticancer Effect

**FIGURE 2:**
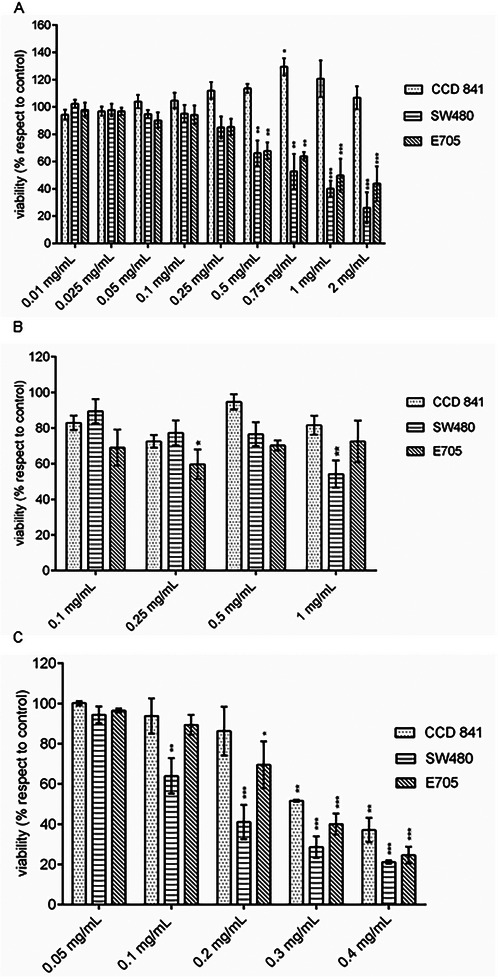
MTT assays to evaluate the effect of the wild strawberry extracts on the cell viability of CCD 841, SW480, and E705 cell lines. Panel (A) shows % cell viability after treatment with total wild strawberry extract; (B) and (C) show the variation of the parameter after treatment with the flowthrough and the eluate fractions, respectively. All histograms are representative of three biological experiments, and % viability mean ± SEM is shown for each concentration tested. ANOVA followed by Dunnett's post hoc test was applied to derive the statistical significance: ***p < 0.001, **p < 0.01, *p < 0.05.

The results of MTT tests are summarized in Figure 2. In particular, the bioactive effects of the wild strawberry total extract on the three colorectal cell lines under investigation are evaluated in Figure 2A. CCD 841 cells were unresponsive to the treatment and showed a significant increase in viability at extract concentrations above 0.75 mg/mL. Cancer cells, on the other hand, decreased their viability starting from a concentration of 0.5 mg/mL. Both cancer cell lines responded to treatment similarly (Figure 2A), although the E705 cell line turned out to be less sensitive than the SW480: the former displayed a viability reduction of nearly 50% when treated with 1 mg/mL extract, while the latter showed a 50% decrease in viability when treated with 0.75 mg/mL extract. SPE loading fraction (flowthrough) did not show any significant effect on either cell line (Figure 2B, C). The concentrations of wild strawberry eluted fraction ranging from 0.1 to 0.4 mg/mL proved to be the most effective; however, a reduction in viability was detected not only for the two cancer cell lines but also for the healthy cell line (Figure 2C). A viability value of about 50% was observed at concentrations of 0.3, 0.1, and 0.2 mg/mL for CCD 841, SW480, and E705 cells, respectively. The following experiments were carried out using the wild strawberry fraction at two different concentrations (i.e., 0.05 and 0.1 mg/mL for SW480 cell line, while 0.1 and 0.2 mg/mL for the E705 one). All the concentrations used on cancer cell lines did not affect CCD 841 cell viability (Figure 2C).

### 3.3 Effect of Wild Strawberry Eluted Fraction on Cancer Cell Oxidative Stress Level

**FIGURE 3:**
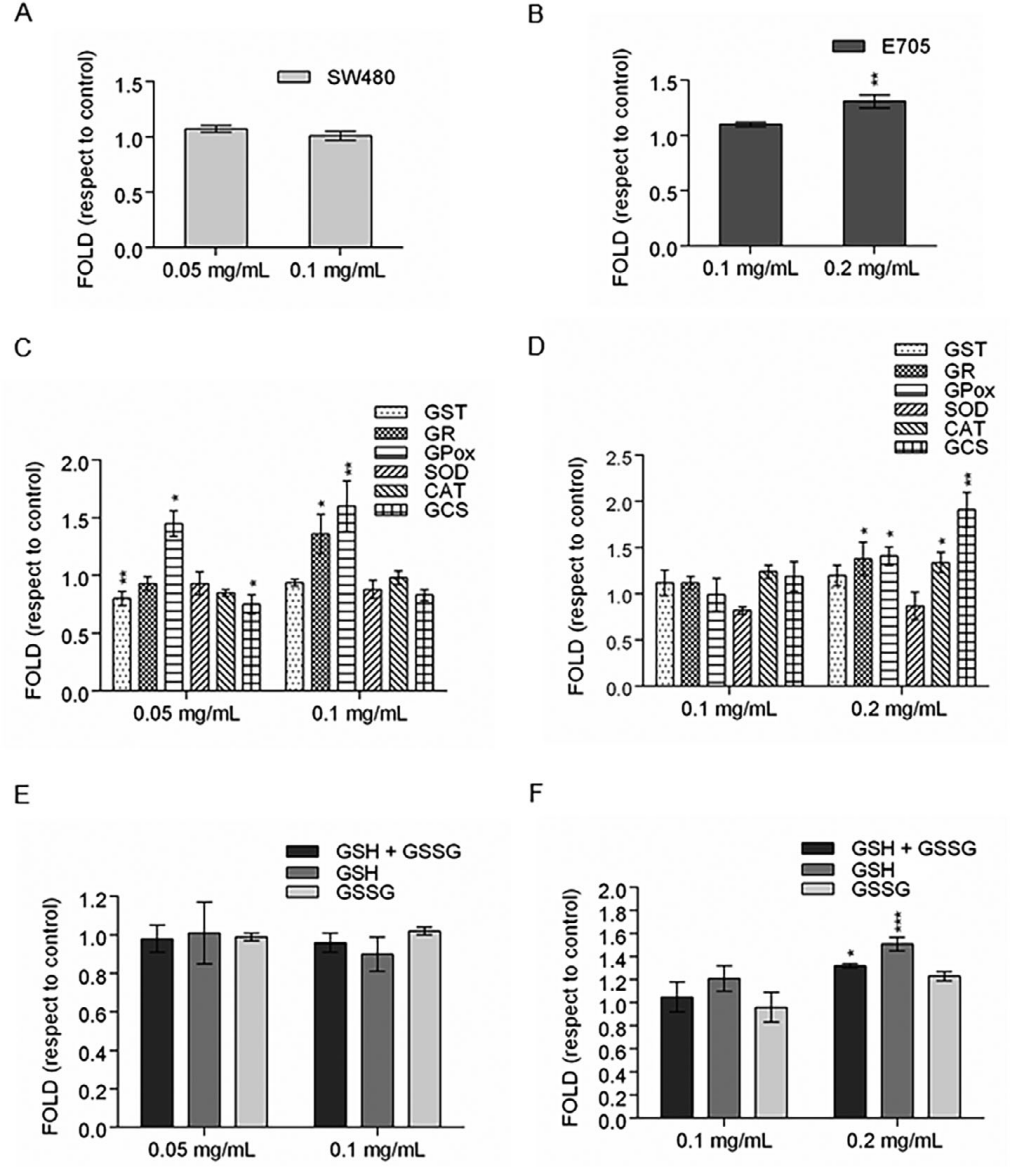
Analysis of reactive oxygen species (ROS) production and major biological systems to detoxify these reactive products. Panels (A) and (B) display the generic ROS production measured by the fluorescence emission of H_2_DCF‐DA in SW480 and E705 cell lines, respectively. Data are expressed as a mean fold value ± SEM of three independent experiments. Panels (C) and (D) show the specific activities of enzymes involved in ROS detoxification for the SW480 and E705 cell lines. Levels of total, reduced, and oxidized glutathione in the SW480 and E705 tumor cell lines are summarized in panels (E) and (F). Data are expressed as a mean fold value ± SEM versus fluorescence of untreated cells. ANOVA followed by Dunnett's post hoc test was applied to derive the statistical significance: ***p < 0.001, **p < 0.01, *p < 0.05.

The analysis of the oxidative stress level of the colorectal cancer cell lines showed an increase in ROS level in the E705 line treated with the wild strawberry eluted fraction at a concentration corresponding to a 50% viability reduction (see Figure 2). No alterations of ROS level were observed in SW480 cells, following treatment with the abovementioned fraction (Figure 3A, B). Figure 3C, D reports the activities of enzymes involved in the oxidative stress defense system in SW480 and E705 cell lines. In the SW480 cell line, an increase in GPox activity was detected under all the experimental conditions. Besides, a significant increase in GR activity after treatment with the highest eluted fraction concentration and a concomitant decrease in GST and GCS activities after treatment with the lowest dose were observed. In the E705 cell line, although the enzyme activities did not vary at the lowest dose of the eluted fraction, most enzymes, notably GCS, increased their activity after treatment with the highest dose (Figure 3D). When reduced (GSH) and oxidized (GSSG) glutathione were assayed, an increase in GSH was detected in E705 cells, compared to control cells, as expected following the increase in GCS activity (Figure 3F). No changes occurred in either glutathione form in SW480 cells (Figure 3E).

### 3.4 Analysis of the Apoptosis Mechanisms

**FIGURE 4:**
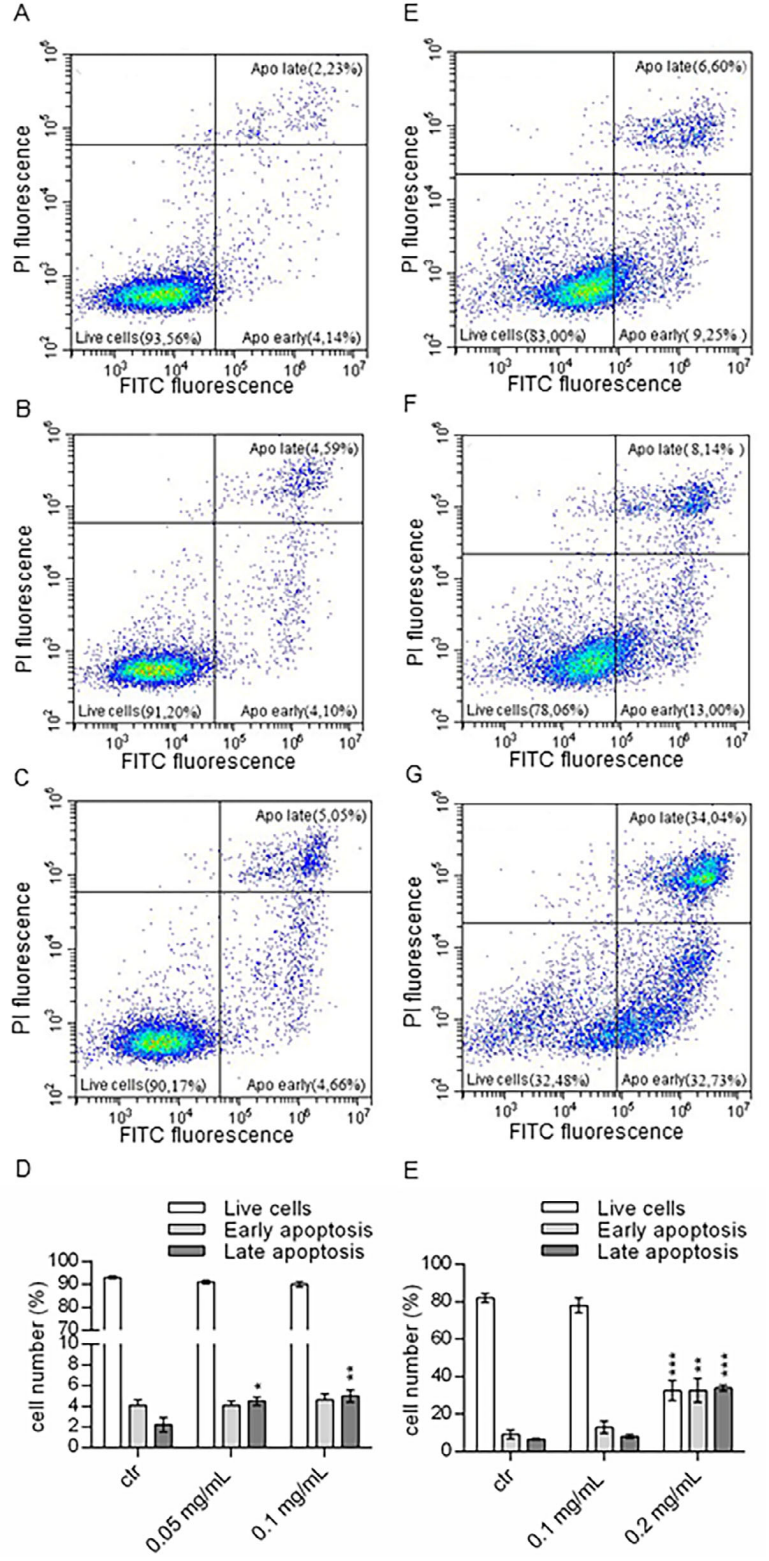
Apoptosis analyses. Two probes were used to study apoptotic mechanisms. Annexin V‐linked fluorophore FITC (X‐axis) recognizes phosphatidylserine under apoptotic conditions; propidium iodide (PI) (Y‐axis) binds DNA in case of plasma membrane damage. Panels (A–C) display the distribution of the cell population in the control and the two treated samples for the SW480 cell line. The location of a single event in each box, represented as a blue dot, is correlated to fluorescence detected for both FITC and PI probes. The dials in each graph indicate the percentage of live cells (bottom left), cells in the early stages of apoptosis (bottom right), and cells in the late stages of apoptosis or necrosis (top right). Analysis of three independent experiments is shown in (D), in which data are defined as a mean fold value ± SEM compared with untreated cells. The same data organization and analyses are depicted in panels (E–H) for the E705 cell line. ANOVA followed by Dunnett's post hoc test was applied to derive the statistical significance: ***p < 0.001, **p < 0.01; *p < 0.05.

Figure 4 reports apoptotic cells analysis for both cancer cell lines, performed through Annexin V/PI protocol. Only a limited increase of the late apoptotic cells content was detected for the SW480 cell line treated with each of the two doses (Figure 4D). The number of cells in late apoptosis increased from 2.23% in untreated cells to about 5% in cells treated with the wild strawberry eluted fraction (Figure 4A–C), suggesting that apoptosis induced by wild strawberry BACs is not responsible for the reduction of cell viability reported in Figure 2. On the contrary, the E705 cell line showed an activation of the apoptotic pathway (Figure 4H) after treatment. Cells in early apoptosis increased from 9.25% in untreated cells to 13.00% and 32.73% after the treatment with 0.1 and 0.2 mg/mL, respectively (Figure 4E–G). The number of cells in late apoptosis/necrosis was five times higher in cells treated with 0.2 mg/mL than in untreated cells.

### 3.5 Wild Strawberry Eluted Fraction Blocks the Cell Cycle of the SW480 Cell Line

**FIGURE 5:**
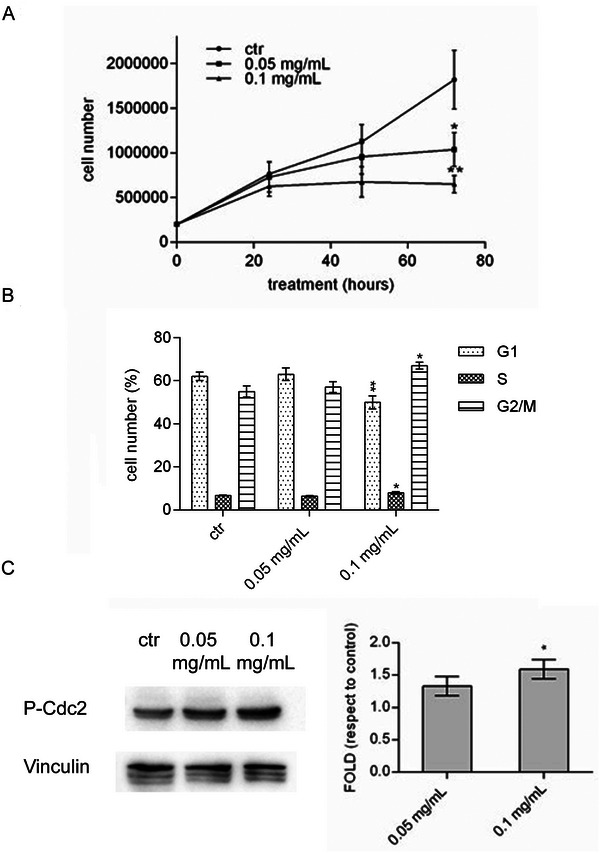
Cell cycle arrest investigation. (A) Kinetic cell proliferation assay. The graph shows the SW480 cells proliferation rate following the reported doses of treatment at three different times: 24, 48, and 72 h. (B) The histogram represents the percentage of SW480 cells at different cell cycle phases following treatment. (C) A representative Western blot conducted on both treated and untreated cells using an anti‐P‐Cdc2 antibody. Vinculin was used as a loading control. Histogram (D) displays the densitometric analysis of three independent experiments. Data are expressed as mean fold value ± SE compared with untreated cells. ANOVA followed by Dunnett's post hoc test was applied to derive the statistical significance: **p < 0.01; *p < 0.05.

To shed light on the decrease in SW480 cell viability in MTT assays, cell cycle progression was investigated through a kinetic cell proliferation assay. Results reported in Figure 5A showed that the wild strawberry eluted fraction is effective in reducing the proliferation rate at both doses; however, the most marked reduction was obtained with the highest dose after 72 h treatment. The analysis of the percentage of cells in the different cell cycle phases revealed that treated cells are blocked in the G_2_/M phase, resulting in a depletion of G_1_ phase (Figure 5B). In agreement with this finding, the phosphorylation level of cdc2 at Tyr15, a critical regulatory condition that does not allow the progression of cell cycle into mitosis, appeared to increase in cells treated with the wild strawberry eluted fraction compared to the control (Figure 5C, D).

## 4 Discussion

Many factors contribute to CRC, the most studied being genetic factors like chromosomal instability, CpG island methylator phenotype, and microsatellite instability, which can be present either together or separately [36]. At the same time, the heterogeneity in CRC incidence demonstrates the involvement of environmental factors, increasing the risk of developing such a pathology; among these, diet appears to be one of the most relevant factors. Many studies have highlighted the preventive role of dietary BACs in counteracting the development of CRC [37]; however, very little is known about the impact of diet on already diagnosed cancer and cancer therapy. Food nutraceutical properties are being studied with increasing interest [11], and BACs have been proposed as coadjuvants for various cancer treatments [38, 39, 40]. Fleshy fruits, such as wild strawberries, are typically known for their antioxidant and antiinflammatory properties [17, 41]; however, their potential against cancer has only recently emerged [42, 43]. The occurrence of many BACs, such as those identified in the present study, is an important indicator of putative nutraceutical properties, including those with potential anticancer activity. BACs and to a lower extent some amino acids are the most represented component in wild strawberry eluted fraction, while in the total extract, a significant content of free sugars (>50% of the dry weight) was detected. Free sugars are probably responsible for the increase in the viability of healthy cells (CCD 841 cell line) under treatment with high doses of extracts since they represent a primary energy source. However, to investigate the nutraceutical properties of the extracts, we focused on the eluted fraction, the only one endowed with anticancer activity. We found a different effect of wild strawberry BACs on the two cancer cell lines used in this study: cell cycle arrest in SW480 cells and apoptosis induction in E705 cells. In SW480 cells, the treatment with wild strawberry eluted fraction does not alter the ROS level; however, the activity of several enzymes involved in the defense against oxidative stress is increased, suggesting that any increase in ROS level, induced by BAC administration, can be effectively counterbalanced at the cellular level. This is also supported by the unchanged glutathione level in both its reduced and oxidized form. This is in accordance with data previously reported by Storz [16], showing that KRAS mutations are normally associated with the upregulation of ROS‐detoxifying enzymes. SW480 cells are characterized by a mutation in the k‐Ras gene making them generally insensitive to treatments with chemotherapeutic agents, like Cetuximab [44, 45]. This drug, which targets the EGFR pathway, does not affect cell survival of the KRAS‐mutated SW480 line, whereas it is widely used for the treatment of CRCs that do not carry KRAS mutations, such as the E705 cell line [46, 47, 48, 49, 50]. In this study, we found that the proliferation of SW480 cells, responsible for very aggressive tumor phenotypes, is significantly slowed down upon treatment with low doses of the eluted fraction of wild strawberry extracts (<100 µg/mL). Several studies have highlighted the sensitivity of mutated forms of K‐Ras to BACs belonging to the family of polyphenols, such as quercetin, chlorogenic acids, and their derivatives, which act as natural inhibitors of the oncogenic variants of the protein [51] and which were found to be highly represented in the wild strawberry eluted fraction. Furthermore, the evidence of a cell cycle arrest supports the hypothesis that the mechanism through which BACs act on the SW480 cell line is mediated by an inhibition of the proliferation signals, which may involve an interplay with the mutated form of K‐Ras. Conversely, the BAC effect on E705 cell lines was found to be more deeply associated to the induction of programmed cell death, as shown by the increase in ROS level promoted by administration of the eluted fraction at the highest dose. The increase in the activity of several enzymes involved in cell defense against oxidative stress, as well as reduced glutathione, cannot counterbalance the increase in ROS level, which eventually leads to apoptosis. A previous work on *Fragaria* x *ananassa* [52] showed cell cycle arrest in G_2_/M together with an anticancer proapoptotic effect of nonextractable strawberry polyphenols on KRAS mutated colorectal cancer HCT116 cells. On the whole, our results confirm previous data obtained by other authors about the role of wild and common strawberries in the regulation of apoptosis and their ability to arrest the cell cycle of different cell types [53, 54, 55, 56, 57].

## 5 Conclusion

Although further studies will be necessary, especially to evaluate the bioavailability of wild strawberry BACs in the gut, this study suggests that diet supplementation with fleshy fruits such as wild strawberries can be a very promising adjuvant both for patients who cannot benefit from EGFR targeted therapy and for those treated with cetuximab to enhance its therapeutic effect. In addition, the lack of toxicity of BACs toward healthy colorectal cells makes them a putative chemopreventive nutrient. Therefore, based on the evidence from this study, their inclusion in the diet may promote both a chemotherapeutic and a chemopreventive effect. In conclusion, in the nutraceutical arena, the role of wild strawberry appears relevant in the prevention of certain forms of colorectal cancer, and future investigations should consider the action of such food on other CRC phenotypes both at in vitro and in vivo level and in the prevention of other types of cancer.

## Conflicts of Interest

The authors declare no conflicts of interest.

### Peer Review

The peer review history for this article is available at https://www.webofscience.com/api/gateway/wos/peer‐review/10.1002/mnfr.70018.

## Supporting information



Supporting Information

## Data Availability

The data that support the findings of this study are available from the corresponding author upon reasonable request.
