# Supplementary material for: Assessment of the Nutraceutical Properties of Wild Strawberry (Fragaria vesca L.) Extracts on Human Colorectal Cell Lines
Source: Mol Nutr Food Res. 2025 Apr 10;69(11):e70018. doi: 10.1002/mnfr.70018 (PMC12128015; doi:10.1002/mnfr.70018)
Supplement: Supplementary file 1 — Supporting Information [file MNFR-69-e70018-s001.docx]

**SUPPLEMENTARY INFORMATION**

**Table S1.** Quantification of the aminoacid content in the different fraction of the wild strawberry extract analyzed at the concentration of 1 mg/mL. Data are expressed as μg of aminoacid per mg of dry weight. The “^*^” indicates the limit of quantification for the different analytes.

| **Amino acid** | **Eluate μg/mg** | **Flowthrough μg/mg** | **Total extract μg/mg** |
| --- | --- | --- | --- |
| **Asp** | 0.567 | 0.119 | 0.383 |
| **Glu** | 1.22 | 0.342 | < 0.01^*^ |
| **Asn** | 7.935 | 0.239 | < 0.078^*^ |
| **Ser** | 0.86 | 0.093 | 0.045 |
| **Gln** | 3.84 | 0.118 | < 0.007^*^ |
| **His** | 0.166 | < 0.078^*^ | < 0.078^*^ |
| **Gly** | 0.213 | 0.132 | 0.025 |
| **Thr** | 0.227 | 0.27 | < 0.078^*^ |
| **Ala** | 3.081 | 0.9 | < 0.047^*^ |
| **Tyr** | < 0.076^*^ | < 0.076^*^ | < 0.076^*^ |
| **Cys** | < 0.781^*^ | < 0.781^*^ | < 0.781^*^ |
| **Lys** | < 0.078^*^ | < 0.078^*^ | < 0.078^*^ |
| **Phe** | 0.313 | 0.432 | < 0.156^*^ |
| **Ile** | 0.093 | < 0.078^*^ | 0.302 |
| **Leu** | 0.053 | 0.072 | < 0.044^*^ |
| **Pro** | 0.028 | 0.022 | < 0.003^*^ |
| **Val** | 0.54 | 0.274 | 0.117 |
| **Met** | < 0.078^*^ | < 0.078^*^ | < 0.078^*^ |
| **Arg** | 0.214 | 1.197 | 0.507 |
| **Trp** | 0.234 | 0.01 | 0.004 |
| **Total** | 19.584 | 4.22 | 1.383 |

**Table S2.** List of the identified metabolites by HRMS in both negative and positive ion currents. Abundance estimations are indicated as peak intensity and quantitation expressed in aglycone equivalent. Rt = Retention time: m/z = mass/charge ratio.

| **m/z** | **Rt** | **Eluate (peak intensity)** | **Flow-through (peak intensity)** | **Total extract (peak intensity)** | **Fragments** | **Adduct** | **Tentative identification** | **Molecular Formula** | **Eluate (mg/g)** | **Reference aglycone** | **Reference** |
| --- | --- | --- | --- | --- | --- | --- | --- | --- | --- | --- | --- |
| **Negative ion mode** | | | | | | | | | | | |
| 629.05 | 4.14 | 5.5E+04 | 1.5E+05 | 6.8E+03 | 615 613 **601** 599 | [M-H]- | Gallotannin | C27H18O18 | 0.57 ± 0.01 | Gallic acid | Sun et al., 2014 |
| 947.04 | 4.17 | 7.2E+04 | 1.3E+05 | 3.5E+03 | **929** 901 883 875 | [M-H]2- | Ellagitannin | C41H24O27 | 0.43 ± 0.02 | Ellagic acid | Sun et al., 2014 |
| 577.2 | 5.05 | 1.0E+05 | 2.6E+04 | 3.4E+04 | 451 425 407 **289** | [M-H]- | Procyanidin Dimer | C30H26O12 | < LOQ (1.62 µg/ml) | Catechin | Sun et al., 2014 |
| 289.07 | 5.47 | 9.1E+05 | 2.2E+05 | 3.1E+05 | 247 245 205 **203** 109 | [M-H]- | Catechin | C15H14O6 | 0.49 ± 0.02 | Catechin | Sun et al., 2014 |
| 325.09 | 6.03 | 1.7E+05 | 4.1E+04 | 5.1E+05 | 285 163 **145** 117 | [M-H]- | Coumaryl hexose | C15H18O8 | 0.61 ± 0.02 | Coumaric acid | Sun et al., 2014 |
| 431.14 | 7.15 | 1.4E+06 | 1.2E+05 | 1.6E+06 | **269** 268 241 147 | [M-2H]- | Pelargonidin-3-O-Glu | C21H21O11 | 1.42 ± 0.01 | Pelargonidin | Sun et al., 2014 |
| 563.16 | 7.04 | 3.1E+05 | 2.7E+04 | 3.1E+03 | 517 359 299 235 **193** 175 | [M-COOH]- | 6′-O-β-D-Glucosyl gentiopicroside | C22H30O14 | 0.26 ± 0.02 | Gentiopicroside | Public spectral libraries |
| 449.11 | 7.54 | 3.9E+05 | 3.2E+04 | 4.5E+05 | 355 329 287 **269** 193 165 | [M-H]- | Astilbin | C21H22O11 | 0.77 ± 0.03 | Taxifolin | Public spectral libraries |
| 935.07 | 8.31 | 1.5E+05 | 1.4E+04 | 3.3E+04 | 463 315 **301** | [M-H]- | Galloyl-bis-hexahydroxydiphenoil (HHDP) glucose | C41H28O26 | 1.22 ± 0.01 | Gallic acid | Sun et al., 2014 |
| 473.17 | 8.62 | 2.0E+05 | 1.6E+03 | 9.8E+05 | **269** 241 225 224 | [M-2H]- | Pelargonidin Acetyl Hexoside | C23H23O11 | 2.2 ± 0.08 | Pelargonidin | Sun et al., 2014 |
| 567.21 | 8.67 | 1.7E+06 | 1.3E+04 | 6.2E+04 | **521** 359 | [M-COOH]- | Methylated Flavonoid Hexoside | C26H34O11 | - # |  | Elshamy et al., 2019 |
| 447.06 | 8.8 | 7.6E+05 | 1.7E+04 | 2.9E+05 | **301** 300 257 229 147 | [M-H]- | Ellagic Acid Rhamnoside | C20H16O12 | 1.09 ± 0.05 | Ellagic acid | Del Bubba et al., 2012 |
| 521.24 | 9.01 | 6.8E+05 | 1.7E+03 | 2.7E+03 | 503 **359** 344 265 | [M-H]- | Tetramethyl Ellagic Acid Hexoside | C26H34O11 | 0.78 ± 0.04^*^ | Ellagic acid | Sun et al., 2014 |
| 447.11 | 9.06 | 3.6E+05 | 1.9E+04 | 4.4E+05 | **301** 300 269 | [M-H]- | Ellagic Acid Rhamnoside | C20H16O12 |  | Ellagic acid | Sun et al., 2014 |
| 301.03 | 9.26 | 1.7E+06 | 3.1E+04 | 1.1E+05 | 284 257 **229** | [M-H]- | Ellagic Acid | C14H6O8 | 11.45 ± 0.21 | Ellagic acid | Del Bubba et al., 2012 |
| 477.12 | 9.57 | 7.8E+05 | 1.8E+04 | 2.9E+06 | **315** 301 300 | [M-H]- | Methyl Ellagic Acid Hexose | C22H22O12 | 3.16 ± 0.10 | Ellagic acid | Public spectral libraries |
| 435.09 | 9.78 | 9.0E+06 | 2.2E+05 | 8.1E+03 | 303 **285** 199 151 | [M-H]- | Taxifolin-3-O-Arabinofuranoside | C20H20O11 | 11.98 ± 0.43 | Taxifolin | Sun et al., 2014 |
| 519.13 | 10.22 | 2.1E+05 | 1.2E+03 | 7.6E+01 | **315** 300 289 195 | [M-H]- | Methyl Ellagic Acid Acetyl Hexose | C23H20O14 | 0.03 ± 0.01 | Ellagic acid | Sun et al., 2014 |
| 447.06 | 10.39 | 7.1E+05 | 5.0E+02 | 2.5E+03 | **315** 300 271 | [M-H]- | Methyl Ellagic Acid Pentose | C20H16O12 | 0.66 ± 0.02 | Ellagic acid | Sun et al., 2014 |
| 447.14 | 11.08 | 2.5E+06 | 5.7E+03 | 3.8E+05 | 285 **284** 255 227 183 | [M-H]- | Kaempferol-3-O-Hexoside | C21H20O11 | 0.76 ± 0.08 | Kaempferol | Sun et al., 2014 |
| 461.13 | 10.84 | 1.6E+07 | 5.8E+04 | 6.0E+05 | **315** 300 271 244 200 | [M-H]- | Methyl Ellagic Acid Methyl Pentoside | C21H18O12 | 14.57 ± 0.02 | Ellagic acid | Sun et al., 2014 |
| 461.13 | 11.15 | 1.1E+06 | 1.9E+03 | 3.5E+04 | **285** 284 255 227 | [M-H]- | Kaempferol-3-O-Glucuronide | C21H18O12 | 0.29 ± 0.01 | Kaempferol | Public spectral libraries |
| 489.16 | 11.58 | 7.9E+05 | 1.2E+03 | 5.2E+05 | 447 337 315 284 **285** 255 | [M-H]- | Kaempferol Acetyl Hexoside | C23H22O12 | 0.66 ± 0.03 | Kaempferol | Del Bubba et al., 2012 |
| 949.16 | 12.08 | 7.3E+05 | 1.3E+04 | 6.5E+04 | 931 895 473 451 **301** | [M-H]2- | Ellagitannin | C44H32O27 | 0.48 ± 0.01 | Ellagic acid | Public spectral libraries |
| 493.28 | 12.26 | 8.6E+05 | 1.0E+03 | 2.3E+03 | **447** 315 301 300 271 161 | [M-COOH]- | Geranyl derivative | C21H36O10 | - $ | Geranyol | Public spectral libraries |
| 549.16 | 13.08 | 7.6E+05 | 9.3E+01 | 5.3E+02 | 455 301 279 **255** 137 | [M-H]- | Liquiritin Apioside | C26H30O13 | 0.66 ± 0.03 | Liquiritin | Public spectral libraries |
| 711.39 | 12.81 13.65 14.39 | 8.2E+04 | 7.9E+01 | 1.5E+05 | **503** 399 343 296 | [M-COOH]- | Arjunglucoside isomers | C36H58O11 | 62.81 ± 2.6 | Asiatic acid | Public spectral libraries |
| 561.2 | 13.98 | 3.5E+05 | 1.8E+01 | 1.2E+02 | **163** 145 119 | [M-H]- | Coumaric Acid Derivative | C28H34O12 | 0.35 ± 0.01 | Coumaric acid | Llorent-Martinez et al., 2015 |
| 593.13 | 14.42 | 2.5E+06 | 2.1E+02 | 3.0E+05 | 447 307 **285** 255 | [M-H]- | Kaempferol-3-O-Coumaoyl-Glucoside | C30H25O13 | 0.65 ± 0.02 | Kaempferol | Sun et al., 2014 |
| 695.4 | 16.32 | 3.8E+06 | 1.8E+02 | 2.9E+05 | 649 **487** 488 | [M-COOH]- | Arjunctin isomer | C36H58O10 | 18.69 ± 2.92 | Asiatic acid | Public spectral libraries |
| **Positive ion mode** | | | | | | | | | | | |
| 188.08 | 3.15 | 2.1E+06 | 1.6E+06 | 4.4E+05 | 188 170 146 143 **118** 115 98 91 77 72 56 | [M-H]+ | Tryptophan | C11H12N2O2 | See Table S1 | Tryptophan | Public spectral libraries |
| 449.13 | 3.7 | 1.4E+07 | 2.2E+06 | 6.7E+05 | 390 **287** 213 137 | M+ | Cyanidin hexoside | C21H20O11 | 2.04 ± 0.08 | Cyanidin-3-O-glycoside | Public spectral libraries |
| 433.12 | 3.9 | 2.2E+07 | 3.5E+06 | 2.2E+07 | **271** 197 121 | M+ | Pelargonidin-3-O-hexoside | C21H21O10 | 1.42 ± 0.01 | Pelargonidin | Spinola et al., 2015 |
| 463.15 | 4.1 | 8.0E+06 | 4.6E+05 | 1.6E+04 | **301** 286 258 230 153 | M+ | Peonidin-3-O-Betagalactoside | C22H22O11 | 1.05 ± 0.05 | Peonidin-3-O-glucoside | Public spectral libraries |
| 519.14 | 4.55 | 6.6E+06 | 1.3E+05 | 1.6E+07 | 301 **271** 201 173 | M+ | Pelargonidin-3-O malonylglucoside | C24H23O13 | 1.28 ± 0.1 | Pelargonidin | Del Bubba et al., 2012 |
| 303.07 | 5.06 | 1.6E+06 | 2.7E+04 | 2.6E+06 | 285 275 **257** 247 229 213 201 191 173 162 145 117 | M+ | Ellagic Acid | C14H6O8 | 11.45 ± 0.21 | Ellagic acid | Public spectral libraries |
| 305.08 | 5.25 | 5.4E+06 | 2.1E+05 | 1.4E+04 | 258 241 213 185 **153** 149 123 111 | [M-H]+ | Taxifolin | C15H12O7 | 15.39 ± 0.05 | Taxifolin | Public spectral libraries |
| 287.07 | 5.44 | 4.0E+06 | 1.7E+04 | 1.3E+06 | **287** 201 153 137 121 98 | M+ | Cyanidin | C15H11O6 | 2.11 ± 0.01 | Cyanidin-3-O-glycoside | da Silva et al., 2007 |
| 317.05 | 5.5 | 1.5E+06 | 2.6E+04 | 6.2E+03 | 302 285 **257** 246 229 218 201 190 173 162 145 117 | [M-H]+ | 3-O-Methylellagic acid | C15H8O8 | < LOQ (2.33 µg/ml) | Ellagic acid | Public spectral libraries |
| 535.14 | 5.78 | 1.4E+06 | 3.3E+03 | 8.3E+05 | **287** 201 98 | M+ | Cyanidin 3-(6''-malonylglucoside) | C24H23O14 | 0.28 ± 0.01 | Cyanidin-3-O-glycoside | da Silva et al., 2007 |
| 595.18 | 7.08 | 1.2E+06 | 1.2E+02 | 1.7E+05 | 549 287 201 165 **147** 119 98 91 | [M-H]+ | 2-arylbenzofuran flavonoid | C31H30O12 | - # | - | Hassimotto et al., 2007 |
| 257.09 | 7.3 | 1.8E+06 | 1.1E+03 | 1.5E+04 | 215 **153** 131 103 | [M-H]+ | Pinocembrin | C15H12O4 | 0.35 ± 0.01 | Pinocembrin | Public spectral libraries |

#, specific aglycone not identified

$, no absorbance in UV/visible without mobile phases interference

^*^ quantitation refers to both compounds due to their similar retention time.

**References**

da Silva, F. L., Escribano-Bailón, M. T., Alonso, J. J. P., Rivas-Gonzalo, J. C., & Santos-Buelga, C. (2007). Anthocyanin pigments in strawberry. *LWT-Food Science and Technology*, *40*(2), 374-382.

Del Bubba, M., Checchini, L., Chiuminatto, U., Doumett, S., Fibbi, D., & Giordani, E. (2012). Liquid chromatographic/electrospray ionization tandem mass spectrometric study of polyphenolic composition of four cultivars of Fragaria vesca L. berries and their comparative evaluation. *Journal of Mass Spectrometry*, *47*(9), 1207-1220.

Elshamy, A. I., Abdallah, H. M. I., El Gendy, A. E. N. G., El-Kashak, W., Muscatello, B., De Leo, M., & Pistelli, L. (2019). Evaluation of anti-inflammatory, antinociceptive, and antipyretic activities of Prunus persica var. nucipersica (nectarine) kernel. *Planta medica*, *85*(11/12), 1016-1023.

Hassimotto, Neuza Mariko Aymoto, Maria Inês Genovese, and Franco Maria Lajolo. "Identification and characterisation of anthocyanins from wild mulberry (Morus nigra L.) growing in Brazil." *Food science and technology international* 13.1 (2007): 17-25.

Llorent-Martínez, E. J., Spínola, V., Gouveia, S., & Castilho, P. C. (2015). HPLC-ESI-MSn characterization of phenolic compounds, terpenoid saponins, and other minor compounds in Bituminaria bituminosa. *Industrial Crops and Products*, *69*, 80-90.

Spínola, V., Pinto, J., & Castilho, P. C. (2015). Identification and quantification of phenolic compounds of selected fruits from Madeira Island by HPLC-DAD–ESI-MSn and screening for their antioxidant activity. *Food chemistry*, *173*, 14-30.

Sun, J., Liu, X., Yang, T., Slovin, J., & Chen, P. (2014). Profiling polyphenols of two diploid strawberry (*Fragaria vesca*) inbred lines using UHPLC-HRMS^n^. *Food chemistry*, *146*, 289-298.
